# Orthogonal Staggered Alignment of Molecular Chains in Aramid Whiskers: A Game‐Changer for Sustainable, High‐Performance Composites

**DOI:** 10.1002/marc.202500919

**Published:** 2026-01-20

**Authors:** Satoshi Okamoto, Justin Llandro, Andrew Gibbons, Mohamed Arfaoui, Daisuke Hashizume, Junji Watanabe

**Affiliations:** ^1^ RIKEN Baton Zone Program TRIP Headquarters Wako Saitama Japan; ^2^ Materials and Structures Laboratory Institute of Integrated Research Institute of Science Tokyo Yokohama Kanagawa Japan; ^3^ Research Planning and Coordination Department Sumitomo Chemical Co., Ltd., Japan Chuo‐ku Tokyo Japan; ^4^ RIKEN Center for Emergent Matter Science (CEMS) Wako Saitama Japan; ^5^ Laboratory for Future Interdisciplinary Research of Science and Technology Institute of Science Tokyo Yokohama Kanagawa Japan

**Keywords:** enhanced surface energy, high‐performance composites, orthogonal chain alignment, polymer single crystal whisker, staggered crystalline architecture

## Abstract

We report the first discovery of polymer single crystal whiskers with a fundamentally unprecedented architecture: prismatic poly(p‐benzamide) (PBA) whiskers with practical dimensions (∼17 µm length, ∼1 µm width, ∼0.5 µm thickness). Within these whiskers, polymer chains are oriented perpendicular to the long axis while maintaining high crystallinity. This morphology diverges from conventional lamellar structures, representing a new class of staggered polymer crystalline architecture where adjacent chains interpenetrate without layered periodicity. The configuration exposes chain ends at whisker surfaces, significantly enhancing dispersibility in organic solvents and polymer matrices due to the high‐energy surface. Composites incorporating these whiskers exhibit uniform microstructures, exceptional thermal stability, and high modulus. PBA whiskers offer a lightweight, organic alternative to traditional inorganic fillers. These findings not only introduce a sustainable strategy but also redefine structural design principles in polymer crystallography for high‐performance materials via self‐organization pathways.

## Graphical abstract

Poly(p‐benzamide) whiskers with orthogonal chain alignment form prismatic single crystals, enabling uniform dispersion and high crystallinity. This architecture enhances composite thermal stability, modulus, and dimensional integrity, offering a sustainable, lightweight alternative to inorganic fillers and redefining polymer crystallography through self‐organized staggered packing.

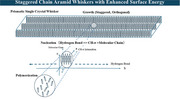

## 1 Introduction

The molecular orientation of polymer chains within crystalline or liquid‐crystalline fibers critically governs their mechanical, thermal, and interfacial properties. Conventional polymer fibers and whiskers—highly crystalline, fiber‐like structures—typically exhibit chain alignment parallel to their long axis [1, 2]. This configuration imparts exceptional axial strength but significantly limits dispersibility and interfacial compatibility, hindering the development of composites with uniform microstructures and tunable properties.

Kevlar fibers, derived from solution spinning of poly(p‐phenylene terephthalamide) (PPTA), exemplify this trade‐off: their highly oriented chains deliver outstanding tensile strength and thermal stability, yet require surface modification to overcome poor dispersibility and interfacial adaptability [3]. Similarly, cellulose nanofibers (CNFs), widely studied as lightweight reinforcing fillers, exhibit strong axial chain alignment and abundant hydroxyl groups that form dense hydrogen‐bonding networks, which restrict dispersion in aqueous and organic media [4]. Poly(p‐oxybenzoate) (POB) whiskers face similar challenges due to their rigid, axially aligned chain structures [5].

In this study, we report poly(p‐benzamide) (PBA) whiskers with a fundamentally distinct molecular orientation: polymer chains aligned perpendicular to the long axis of the crystal [6]. This orthogonal configuration diverges sharply from conventional lamellar and axially aligned systems, introducing a new class of staggered crystalline architecture. We investigate the internal chain arrangement, crystallinity, and morphology of these whiskers, and demonstrate how this unique orientation enhances dispersibility, interfacial integration, and composite performance. These findings offer a new paradigm for polymer crystallography and provide a sustainable strategy for designing high‐performance organic fillers.

## 2 Results

### 2.1 Morphology and Crystallinity of PBA Whiskers

Single‐crystalline poly(p‐benzamide) (PBA) whiskers were successfully synthesized via phase‐change polymerization using monomers derived from 4‐acetamidobenzoic acid [5]. The reaction conducted at 340°C with a monomer concentration of 1 wt.% yielded a whisker with high purity, achieving a yield of 93% after 8 h.

**FIGURE 1:**
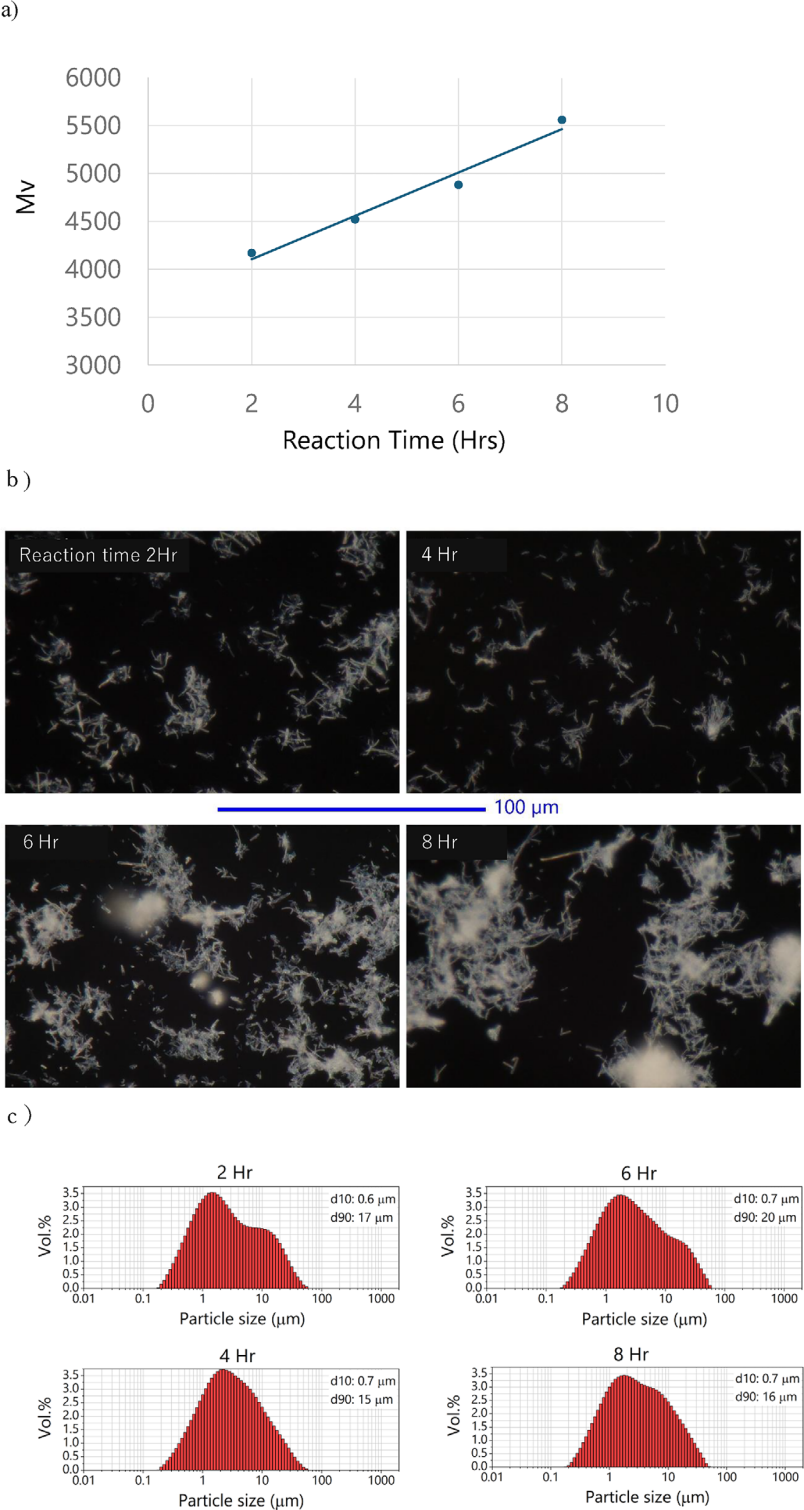
Morphology and molecular characteristics of PBA whiskers. a) Molecular weight vs. reaction time. b) POM images showing anisotropic growth. c) Particle size distribution by laser diffraction.

The viscosity‐average molecular weights of polymers constituting the whiskers were estimated from the intrinsic viscosity measurements performed in solutions of 96.4% sulfuric acid at 25°C, using the established Mark–Houwink equation: [7] [η] = 1.9 × 10^−^^7^  Mv^1.7^. The molecular weight increased linearly from 4000 to 5500 as the reaction time extended from 2 to 8 h (Figure 1a), indicating effective and controlled polymer growth.

**FIGURE 2:**
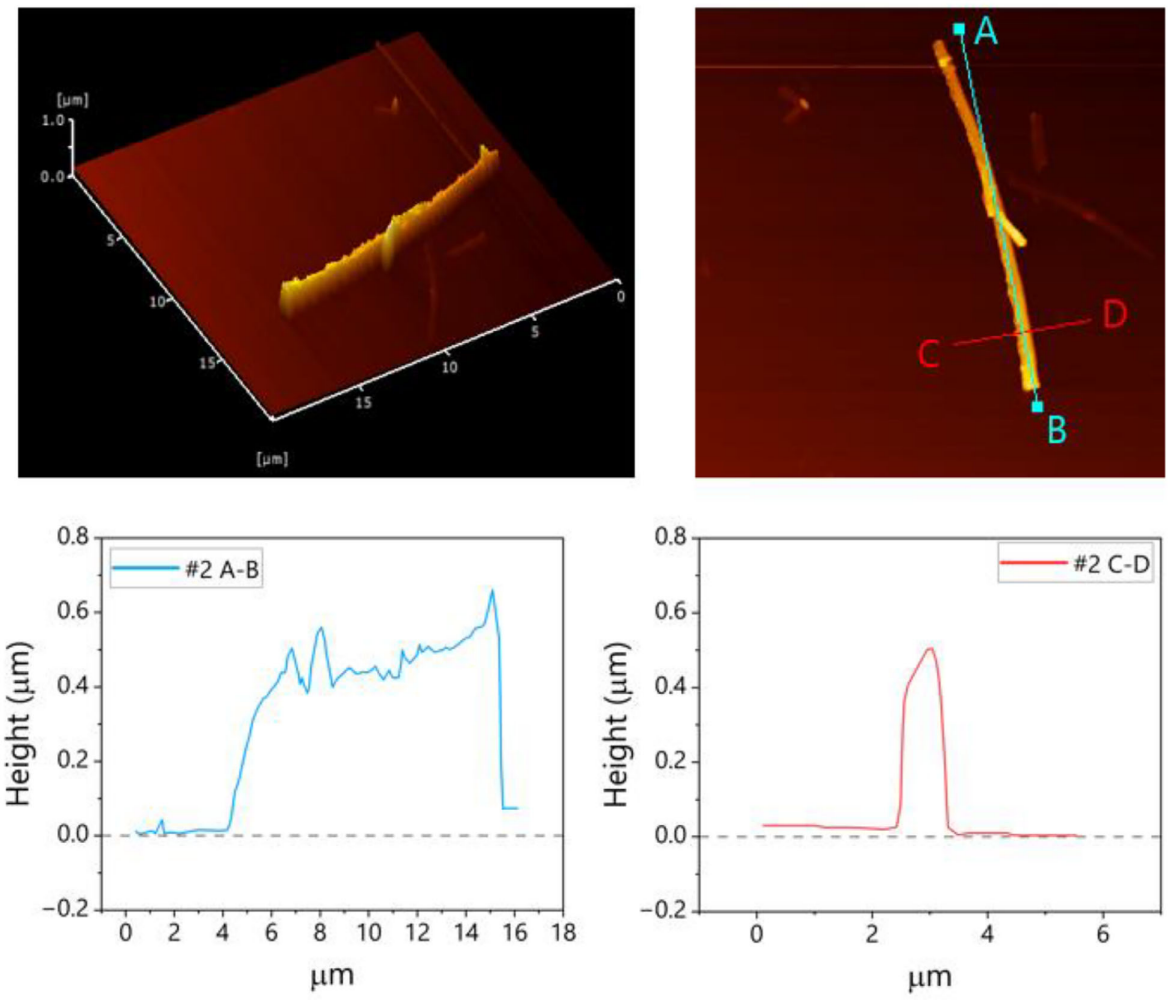
AFM image of a single PBA whisker synthesized at 340°C for 2 h, showing prismatic morphology (length ∼17 µm, width ∼1 µm, thickness ∼0.5 µm).

Polarized optical microscopy (POM) revealed that whiskers synthesized under varying reaction durations exhibited high aspect ratios, indicative of anisotropic crystal growth (Figure 1b). No significant change in aspect ratio was observed between reaction times of 2 and 8 h. In general, particle size distribution measurements by laser diffraction provide d10 and d90 values, which represent the particle diameters below which 10% and 90% of the total particle volume are found, respectively. The particles investigated in this study exhibit a rod‐like morphology, and in laser diffraction measurements, the obtained particle sizes may differ from the actual geometric dimensions due to the influence of particle shape and orientation. However, it is known that for rod‐shaped particles, d90 tends to correspond to the representative value of the particle's long axis, while d10 corresponds to that of the short axis. Laser diffraction analysis further quantified the whisker dimensions, showing average lengths of 15–20 µm (D90) and diameters of 0.6–0.7 µm (d10) (Figure 1c). The d90 and d10 values obtained by laser diffraction were consistent with the particle dimensions observed by POM, confirming the formation of uniformly elongated whiskers across all samples, regardless of reaction time. Furthermore, to investigate the crystal form in greater detail, atomic force microscopy (AFM) was performed on a representative sample of PBA whiskers synthesized at 340°C for 2 h, corresponding to the particle dimensions indicated by d10 and d90 values from laser diffraction (Figure 2).

The whiskers exhibit a well‐defined prismatic morphology with dimensions of approximately 17 µm in length, 1 µm in width, and 0.5 µm in thickness. The density of the whiskers was measured to be 1.47 g/cm^3^, closely matching that of Kevlar fibers [8]. This high density reflects the compact and highly ordered molecular architecture of the PBA whiskers, suggesting a crystalline packing efficiency comparable to that of high‐performance aramid materials.

### 2.2 Orthogonal Chain Alignment Confirmed by Single Crystal Structural Analysis

**FIGURE 3:**
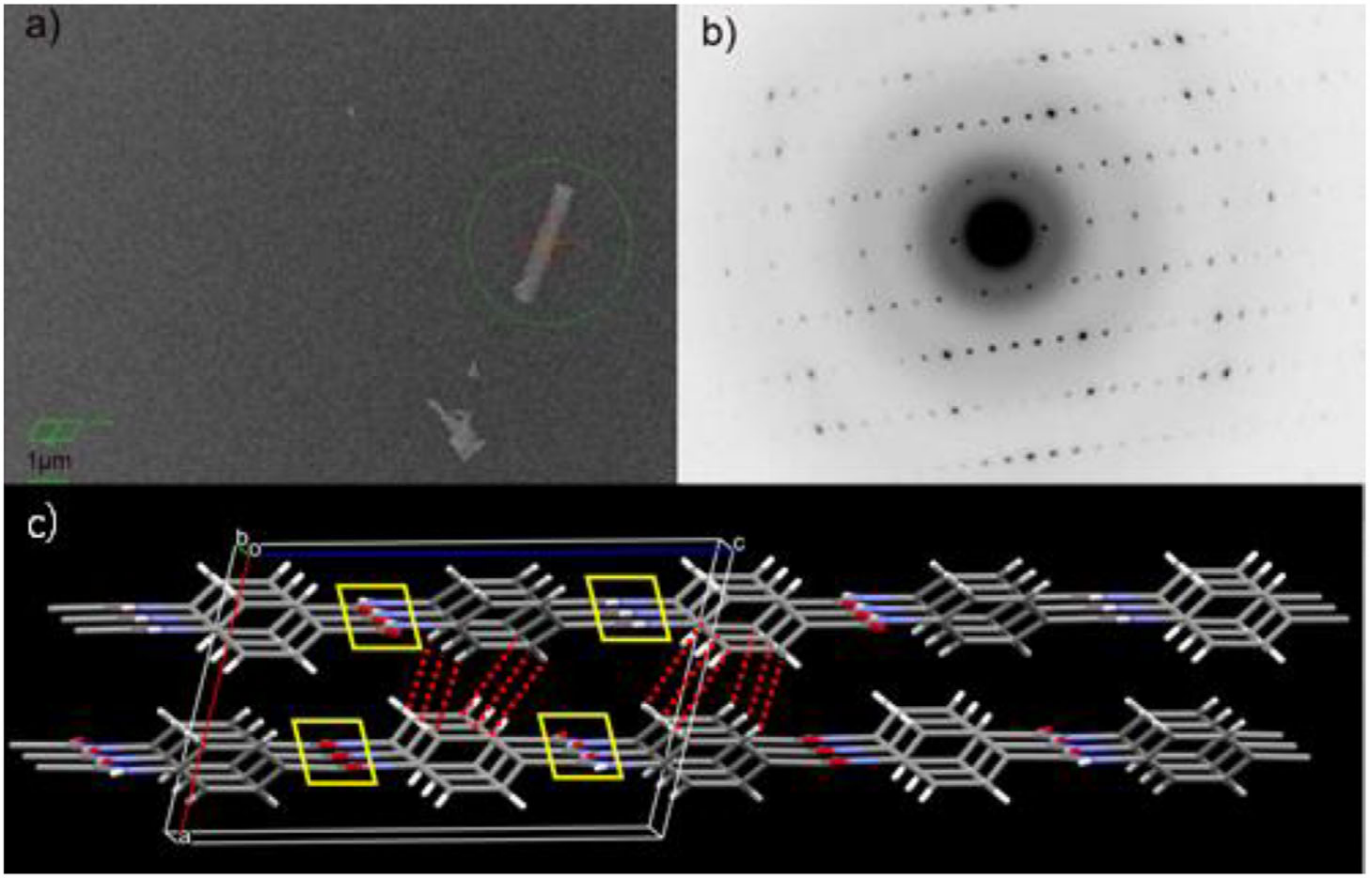
Single crystal structural analysis of PBA whiskers of 8Hr reaction. a) TEM image of a PBA whisker acquired at zero tilt (scale bar: 1 µm), showing a thin prismatic crystal highlighted by a green circle. b) Electron diffraction pattern of the same PBA crystal at zero tilt, confirming its single‐crystalline nature. c) Crystal structure of PBA whiskers. The structure includes two crystallographically independent molecular chains aligned parallel to the *c*‐axis. The yellow boxes and red dotted lines indicate the intermolecular N─H…O hydrogen bonds and C─H…π interactions, respectively.

Microcrystal electron diffraction (MicroED) [9, 10, 11] was employed to determine the crystal structure and molecular orientation of the poly(p‐benzamide) (PBA) whisker. A real‐space image of a PBA whisker, obtained via transmission electron microscopy (TEM) at zero tilt, is shown in Figure 3a. The corresponding electron diffraction pattern (Figure 3b) confirms the single‐crystalline nature of the PBA whisker.

Diffraction data were collected from four individual PBA prismatic single crystals, each covering approximately 80°rotation. The merged dataset achieved 0.94 completeness with *R*_int_ = 0.0170. The crystallographic parameters were determined to be: *a* = 7.977(3) Å, *b* = 5.3588(10) Å, *c* = 13.053(2) Å, *β* = 104.25(2)°, and *V* = 540.9(3) Å^3^, *Z* = 4, *D*_X_ = 1.463 g/cm^3^, monoclinic, *Pc* space group. [12] with a reliability factor of *R*(*F*) = 0.1008 for 1787 reflections with *I* > 2σ(*I*). The 3D crystal structure is illustrated in Figure 3c, revealing two crystallographically independent molecular chains traversing the monoclinic unit cell, aligned parallel to the *c* axis. The neighboring molecular chains are interconnected by N─H…O hydrogen bonds and C─H…π interactions along the *b*‐ and *a*‐axes, respectively.

**FIGURE 4:**
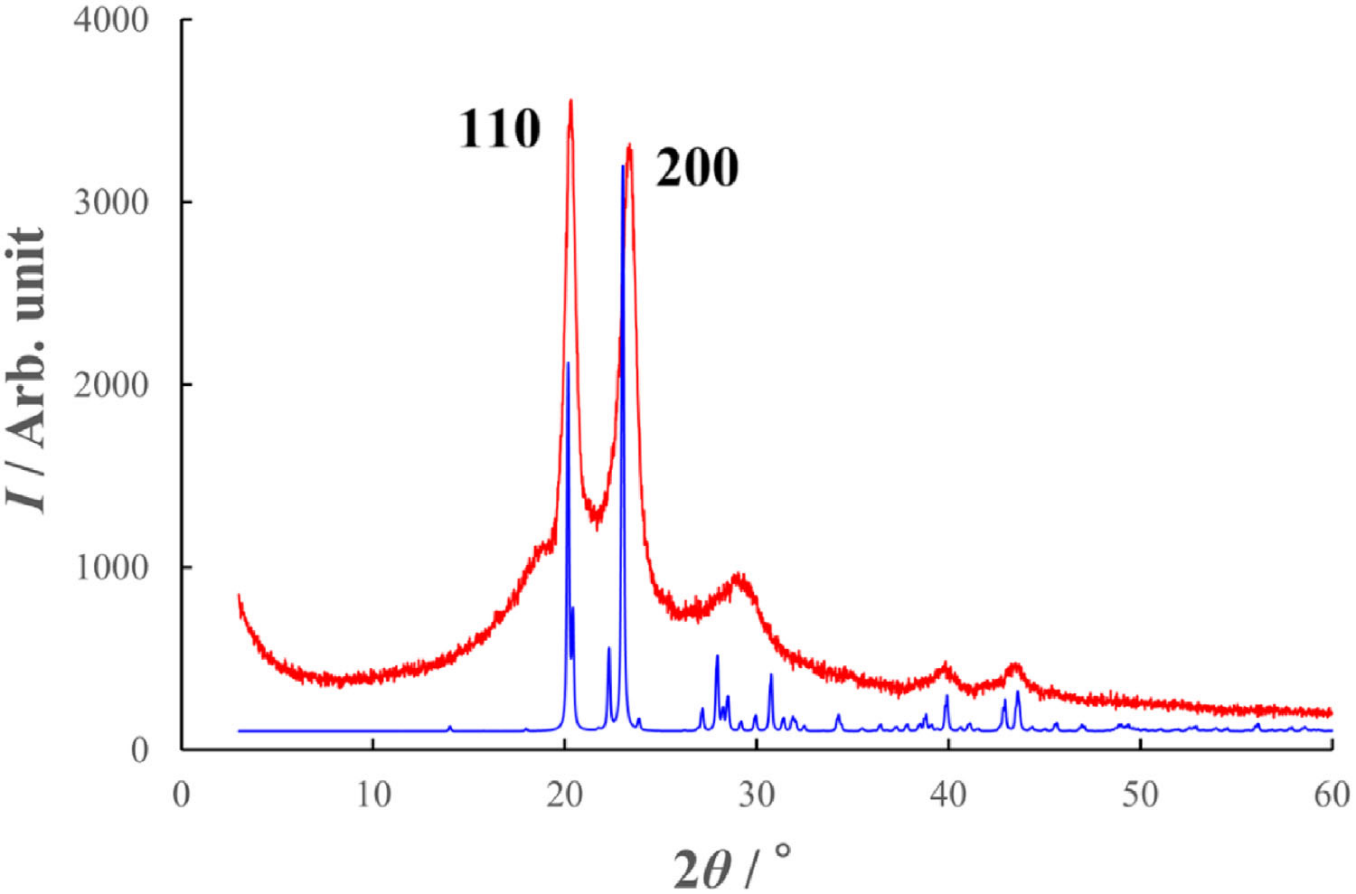
Powder X‐ray diffraction (PXRD) analysis of PBA whiskers. Experimental PXRD pattern of PBA whisker powder (red line) compared with the simulated diffraction pattern calculated from the Micro ED‐derived crystal structure (blue line). The close agreement between the two patterns confirms the structural consistency across characterization techniques.

Complementary X‐ray diffraction measurements are presented in Figure 4. The experimental powder X‐ray diffraction (PXRD) pattern exhibits sharp reflections corresponding to the (110) and the (200) planes, further confirming the single‐crystalline nature of the PBA whiskers [6].

**FIGURE 5:**
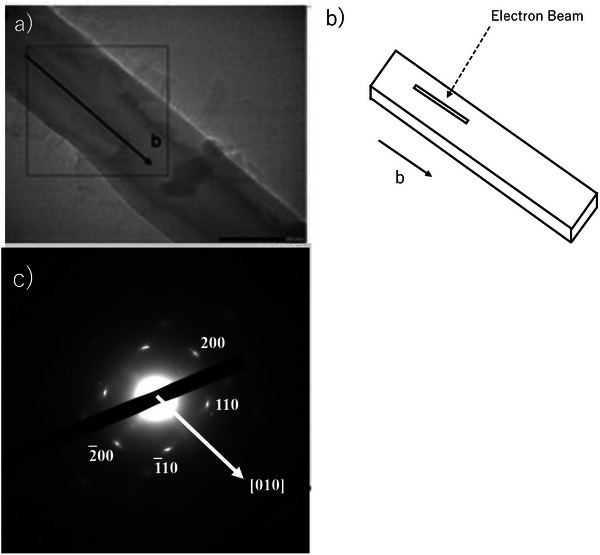
Crystallographic orientation of PBA whiskers revealed by transmission electron microscopy (TEM) and selected‐area electron diffraction (SAED). a) Bright‐field TEM image of a PBA whisker synthesized at 340°C for 2 h. The arrow indicates crystal fiber growth along the *b*‐axis. b) Schematic illustration of lamellar orientation relative to the incident electron beam, corresponding to the configuration observed in a). c) Indexed SAED pattern recorded with the incident beam aligned parallel to the [001] zone axis. Several Bragg reflections are indexed, confirming monoclinic symmetry and high crystallinity.

Notably, the simulated diffraction pattern derived from the Micro ED structure closely matches the experimental PXRD data, indicating structural consistency across techniques. Additional TEM imaging of thin PBA whiskers is shown in Figure 5a. Several whiskers appear tilted relative to the electron beam, exposing their *c*‐axis and revealing a nearly uniform orientation among adjacent crystals. Most whiskers lie flat on the carbon support film, with their major face perpendicular to the beam (Figure 5b). A similar configuration is depicted in Figure 5c, where the electron beam is oriented perpendicular to the long axis of the prismatic whisker.

The selected area electron diffraction (SAED) pattern obtained from the rectangular region marked in Figure 5a is presented in Figure 5c. The indexed reciprocal lattice confirms monoclinic symmetry oriented along the [001] *c*‐axis. Strong reflections corresponding to the (110) and (200) planes indicate high crystallinity and a preferred orientation along the [001] direction. These results suggest that the whisker crystals grow longitudinally along the *b*‐axis, as illustrated in Figure 5a,c.

This orthogonal chain alignment contrasts sharply with the axial orientation typically observed in conventional polymer fibrils and whiskers, such as cellulose nanofibers (CNFs) [13], solution‐spun PPTA fibers like Kevlar [8], and POB whisker [14]. The ability to achieve such alignment through crystallization rather than mechanical processing represents a significant structural innovation in polymer design.

### 2.3 Thermal Stability and Dimensional Integrity of PBA Whiskers

**FIGURE 6:**
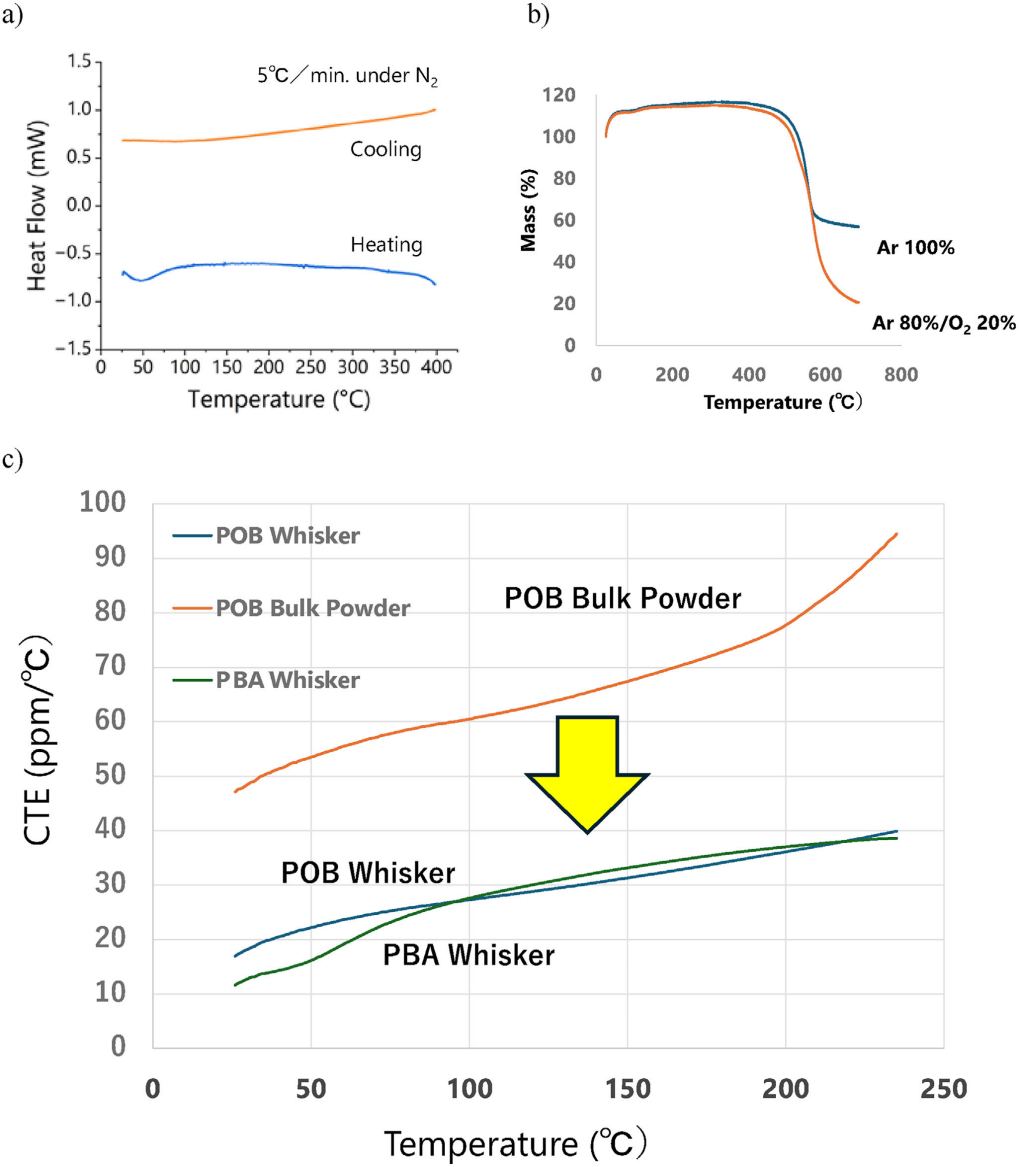
Thermal properties of PBA whiskers of 8Hr reaction. a) DSC profile at 5°C/min scanning rate. b) TGA curves at 10°C/min increasing rate. c) CTE comparison of PBA whisker with POB bulk powder and whisker.

Differential scanning calorimetry (DSC) revealed no glass transition or melting behavior up to 400°C, indicating a highly stable crystalline phase (Figure 6a). Thermogravimetric analysis (TGA) showed decomposition onset at ∼450°C. Residual mass at 600°C exceeded 60% under argon and ∼20% under oxidative conditions (Figure 6b), demonstrating excellent thermal and oxidative stability.

Thermal expansion was measured using a high‐resolution push‐rod dilatometer equipped with a linear variable differential transformer (LVDT) [15]. Powder samples were horizontally placed on the holder, and full‐range calibration ensured accurate determination of the coefficient of thermal expansion (CTE). The obtained CTE profiles are shown in Figure 6c. For comparison, CTE values of PBA whiskers were evaluated alongside bulk‐polymerized POB (Sumitomo Chemical Co., E101) and POB whiskers synthesized via established protocols [5]. PBA whiskers exhibited exceptionally low CTE—less than 25 ppm/°C in the 25°C–100°C range—comparable to POB whiskers and significantly lower than bulk POB polymer. This low thermal expansion is attributed to the rigid aromatic backbone and intermolecular‐chain hydrogen bonding of the PBA whisker, contributing to its dimensional stability under thermal stress.

### 2.4 Humidity Stability of PBA Whiskers

In addition to thermal robustness, the moisture resistance of PBA whiskers dried under vacuum was evaluated by measuring water uptake after 24 h exposure at 23°C and 50% relative humidity [16]. The equilibrium water absorption of PBA whiskers was less than 0.01 wt.% (Figure S1), comparable to POB whiskers and significantly lower than that of cellulose nanofibers under identical conditions. This low hygroscopicity highlights the environmental durability and dimensional precision of PBA whiskers, making them suitable for applications requiring long‐term stability under ambient conditions.

### 2.5 Dispersion in Polymer Matrices

**FIGURE 7:**
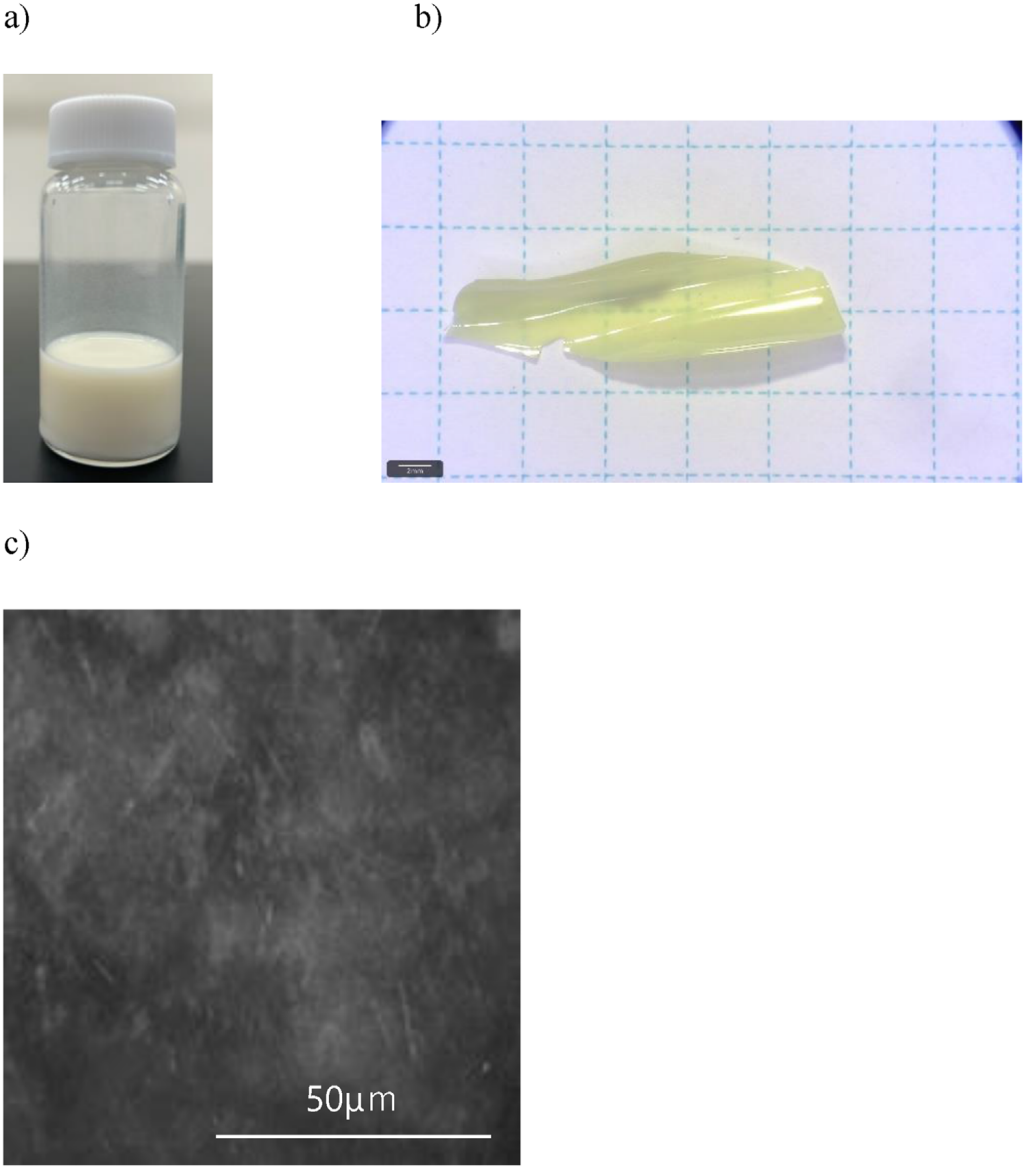
Dispersion behavior of PBA whiskers synthesized with an 8 h reaction time in polymer matrices. a) Homogeneous dispersion of 1 wt.% PBA whiskers in isopropanol (IPA), demonstrating compatibility with polar solvents. b) Composite film of polyethersulfone (PES) containing 10 wt.% PBA whiskers after solvent evaporation, with a thickness of 50 µm. c) Transmission polarizing microscopy image of the PES/PBA composite film (10 wt.%, 50 µm), showing uniform distribution of PBA whiskers without aggregation.

It is noteworthy that the PBA whiskers synthesized here can disperse uniformly in polar organic solvents such as isopropanol (IPA) (Figure 7a), acetone, dimethylformamide (DMF) and hexafluoroisopropanol (HFIP). Owing to this property, it can be utilized as a dispersant in polymer‐based materials. Two representative examples are presented below. To assess dispersibility, PBA whiskers were incorporated into polyethersulfone (PES; SUMIKAEXCEL 4100P) dissolved in dimethylformamide (DMF) at 10wt%. Upon addition of 10wt% PBA whiskers relative to PES content and stirring at 400 rpm, a homogeneous dispersion was readily obtained. By the simple solvent evaporation, the composite film of 50 µｍ‐thickness is homogeneous observed by POM in Figure 7b. Transmission polarizing microscopy image (Figure 7c) exhibited uniform whisker distribution with no observable aggregation.

Similar results were obtained using nylon 6 dissolved in hexafluoroisopropanol (HFIP), demonstrating the compatibility of PBA whiskers with diverse polymer matrices. In contrast, POB whiskers failed to disperse uniformly under identical conditions, resulting in severe segregation and preventing the formation of homogeneous composite films.

### 2.6 Mechanical and Thermal Performance of Composites

**FIGURE 8:**
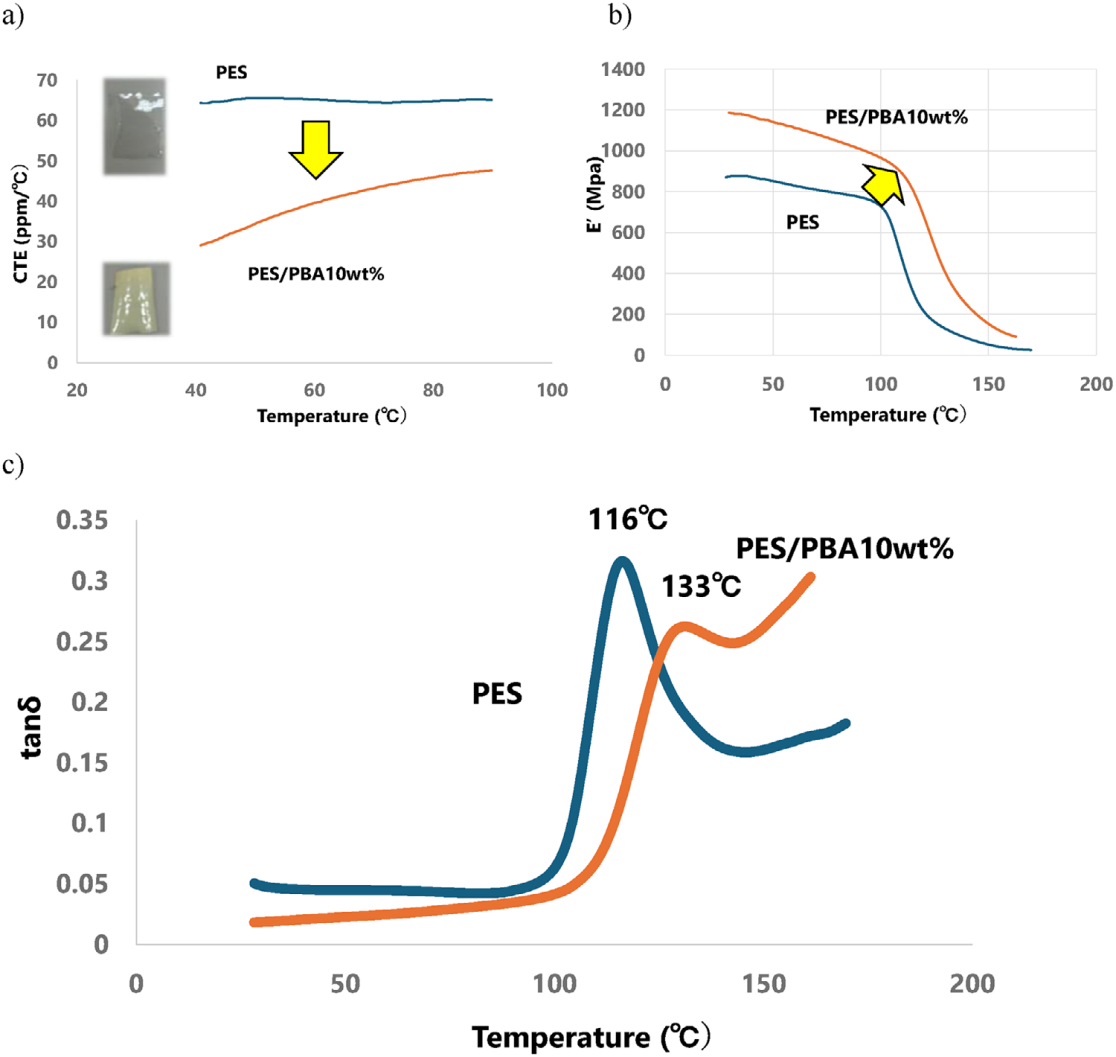
Comparison of mechanical and thermal performance between pristine PES and PES/PBA whisker composite films (10 wt.%, 50 µm thickness). a) Coefficient of thermal expansion (CTE) profiles showing a ∼50% reduction in CTE upon incorporation of PBA whiskers. b) Dynamic mechanical analysis (DMA) curves indicate increased storage modulus and elevated glass transition temperature (Tg) in the composite. c) Tan δ profiles exhibiting a single peak for both films, suggesting uniform phase behavior and strong interfacial compatibility.

The incorporation of PBA whiskers into PES matrices significantly enhanced composite performance. Linear thermal expansion measurements revealed that adding 10 wt.% PBA whiskers reduced the coefficient of thermal expansion (CTE) of PES by approximately 50% compared to pristine PES films (Figure 8a), indicating improved dimensional stability.

Dynamic mechanical analysis (DMA) showed a marked increase in storage modulus and glass transition temperature (Tg) upon whisker addition (Figure 8b), reflecting enhanced mechanical rigidity and thermal resistance. Tan δ profiles exhibited a single peak for both pristine and composite films (Figure 8c), suggesting no segregation and confirming favorable interfacial interactions between PBA whiskers and the polymer matrix.

### 2.7 Studies as a Crystal Nucleating Agent

**FIGURE 9:**
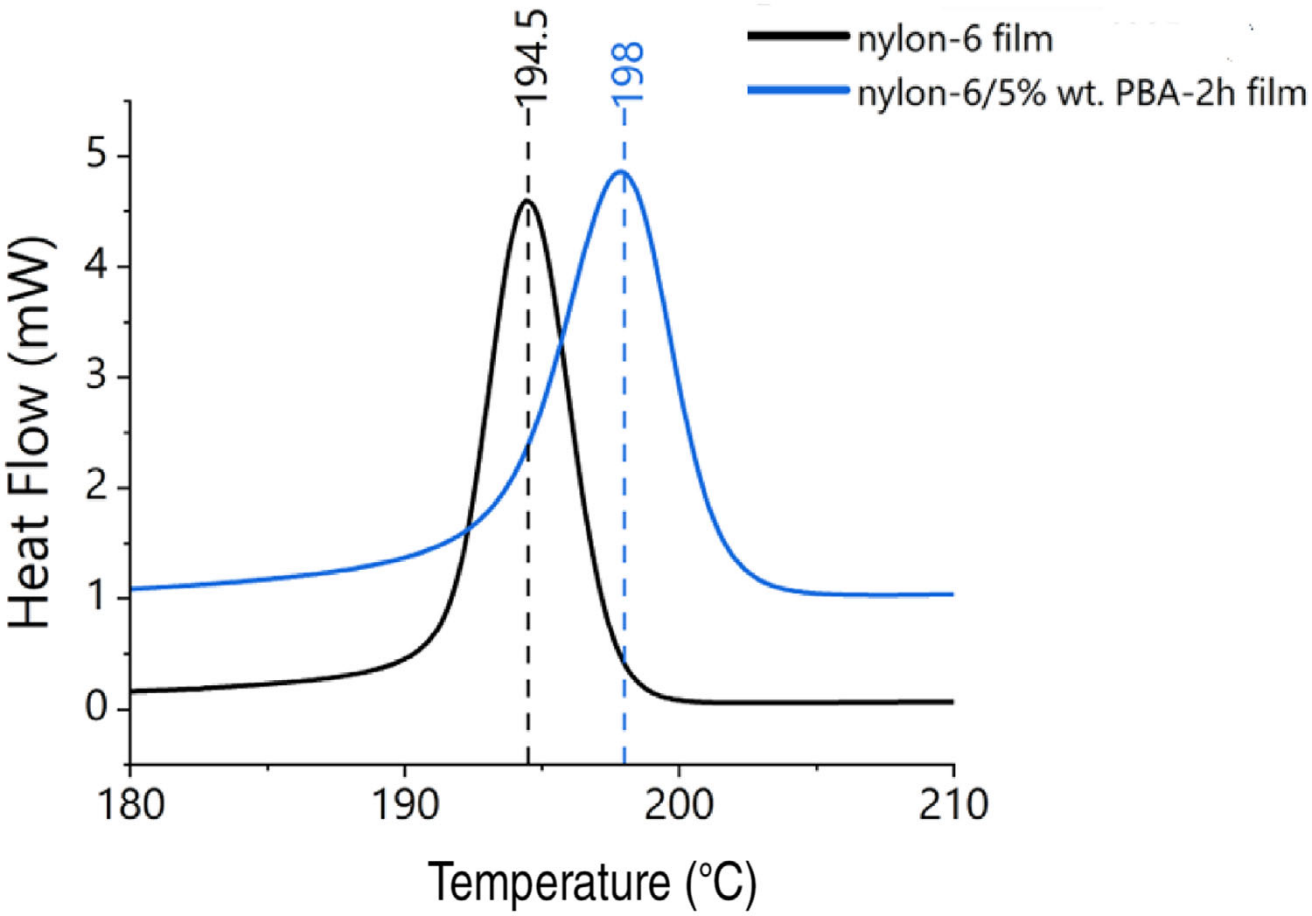
DSC thermograms illustrating the nucleation effect of PBA whiskers on nylon 6 crystallization. The black curve represents neat nylon 6, while the blue curve corresponds to nylon 6 containing 5 wt.% PBA whiskers synthesized with a 2 h reaction time. Data were collected during cooling at a rate of 5°C/min, showing an elevated crystallization onset temperature in the composite.

Takayanagi et al. [17] have reported that PBA acts as a crystallization agent for nylon 6. Figure 9 shows the results of a film obtained by dispersing 5 wt.% nylon 6 with PBA whiskers synthesized by an 8 h reaction. The film was melted at 250°C, and then the temperature was lowered at a rate of 5°C/min. The crystallization temperature was then measured by DSC.

As previously reported, the crystallization onset temperature was found to be approximately 5°C higher than that of a sample containing no PBA. These results indicate that PBA whiskers also function as a crystal nucleating agent for nylon 6.

## 3 Discussions

As shown in Figure 2, the PBA whiskers exhibit a distinct prismatic morphology. This is consistent with the crystalline characteristics reported by Kimura et al., [6] however, the whiskers obtained in this study show a significantly higher aspect ratio and uniformity. This improvement is attributed to the use of a deacetylation polycondensation method, rather than conventional direct polymerization or condensation via acid chlorides typically employed in aramid synthesis, demonstrating the reproducibility and robustness of the present synthetic approach.

The anisotropic growth of these whiskers can be rationalized by the hierarchy of intermolecular interactions: hydrogen bonding along the *b*‐axis, CH/π interactions along the *a*‐axis, and weaker end‐chain interactions along the *c*‐axis (Figure 3c). This energetic gradient (b >> a > c) drives the formation of elongated, high‐aspect‐ratio crystals.

**FIGURE 10:**
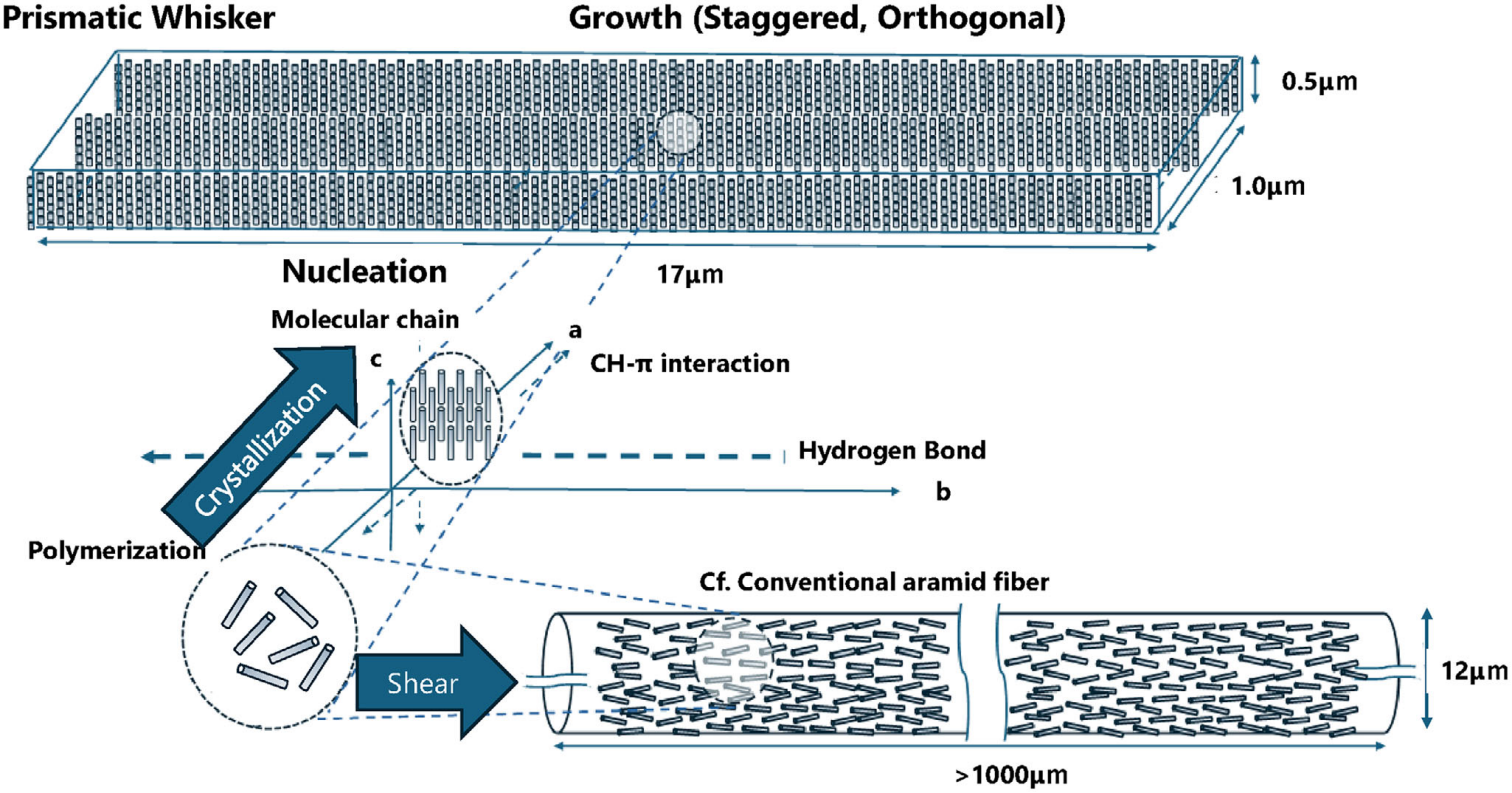
Schematic comparison of molecular packing in PBA whiskers and conventional aramid fibers.

Furthermore, since transmission electron microscopy (TEM) revealed the absence of the lamellar structure typically observed in Kevlar fibers [18], we investigated the internal chain arrangement of PBA whiskers in greater detail. Small‐angle X‐ray scattering (SAXS) was performed on PBA samples polymerized at 340°C for 8 h. Based on the viscosity‐average molecular weight (∼5000, corresponding to approximately 30 repeat units) and an estimated extended chain length of 0.64 nm per unit, a lamellar structure would be expected to exhibit a periodicity of around 20 nm. However, no periodic peaks were observed in the range of 2–50 nm (Figure S2). Considering that no amorphous phase was detected by PXRD (Figure 4), this absence of lamellar ordering suggests that the polymer chains do not adopt a layered configuration, but instead crystallize densely in a non‐periodic, possibly staggered arrangement (Figure 10). The exposed chain ends on the whisker surfaces may offer reactive sites for chemical modification, which could be exploited to improve interfacial compatibility with various matrix materials. Future studies should explore such strategies to enhance composite performance.

To the best of our knowledge, such a structural motif is unprecedented among polymer single crystals. Notably, this unique architecture may originate from the presence of a lyotropic liquid crystalline phase during polymerization. As a result, strong hydrogen bonding and the intermediate liquid‐crystalline phase may cooperatively facilitate chain alignment and interdigitation, leading to the formation of a single‐crystal morphology in which polymer chains adopt a staggered packing motif with their main chain axes oriented orthogonally to the crystallographic growth direction.

In contrast to POB whiskers [19], which typically grow radially from defect sites and exhibit significant chain entanglement, PBA whiskers display linear growth and minimal entanglement. This is attributed to the rigidity of the aromatic backbone. Structurally, PBA shares its backbone with Kevlar [20], yet differs fundamentally in chain orientation. While Kevlar fibers align axially via lyotropic liquid crystal spinning, PBA whiskers exhibit orthogonal, staggered chain alignment.

Despite this unconventional configuration, they exhibit high thermal stability, low thermal expansion, and minimal water uptake compared to cellulose nanofiber data [21, 22, 23] as a conventional eco‐friendly filler (Table S1).

Preliminary mechanical assessments suggest sufficient modulus for engineering applications. Moreover, their anisotropic structure enables directional tuning of thermal conductivity in films and sheets—an elusive property in polymer composites.

Beyond their mechanical and thermal properties, PBA whiskers exhibit second harmonic generation (SHG), indicative of a non‐centrosymmetric crystal structure with a polar *c*‐axis, as previously reported for polymer films and fibers, including POB [24, 25, 26]. Although the SHG intensity is approximately one‐fourth that of POB whiskers due to the extremely thin polar c‐axis (∼0.5 µm), planar alignment of the whiskers is expected to enhance the nonlinear optical response, thereby enabling potential applications in piezoelectric and nonlinear optical devices.

Additionally, PBA whiskers act as nucleating agents for nylon 6 [17], as demonstrated by DSC (Figure 9), enhancing crystallization and mechanical properties of the matrix. This dual functionality—reinforcement and nucleation—positions PBA whiskers as valuable additives for recycled polymers, where property degradation is a persistent challenge.

## 4 Conclusion

In this study, we demonstrated the successful integration of single‐crystalline poly(p‐benzamide) (PBA) whiskers with orthogonally oriented, staggered chains into polymer composite systems. This unique molecular architecture enables uniform dispersion, strong interfacial interactions, and tunable anisotropy, overcoming limitations associated with conventional axially aligned polymer whiskers. The resulting composites exhibit enhanced dimensional stability, mechanical strength, and thermal resistance.

Furthermore, the use of lightweight organic crystalline fillers such as PBA whiskers offers a sustainable alternative to traditional inorganic reinforcements. Given the significant energy demands of the transportation sector, the adoption of such materials could contribute to substantial reductions in fossil fuel consumption and associated emissions [27]. The potential for biosourced synthesis [28, 29] of PBA adds a critical sustainability dimension, positioning these composites as promising candidates for carbon‐neutral or even carbon‐negative material systems. This topic will be elaborated in a forthcoming publication.

Traditionally, polymer chain alignment has been achieved primarily through external forces such as mechanical shear during spinning [2, 30, 31, 32]. However, by harnessing the intrinsic self‐organizing capability of polymer chains, we have demonstrated that even simple conventional polymers can exhibit such highly functional and structurally sophisticated morphologies through crystallization. The discovery of staggered chain packing and orthogonal c‐axis orientation—structural motifs previously limited to inorganic and 2D materials—redefines the principles of polymer crystallography and opens new avenues for the design of multifunctional, sustainable, and high‐performance polymeric materials.

## 5 Methods

### 5.1 Synthesis of PBA Whiskers

Poly(p‐benzamide) (PBA) whiskers were synthesized via phase‐change polymerization using 4‐acetamidobenzoic acid as the monomer. The reaction was conducted under controlled thermal conditions to promote crystal growth. The resulting whiskers were collected by filtration, washed thoroughly with acetone, and dried under vacuum for 10 h at 200°C.

### 5.2 Molecular Weight Estimation

PBA whiskers synthesized under different reaction times were dissolved in concentrated sulfuric acid (96.4%), and the intrinsic viscosity was measured at 25°C using a Ubbelohde viscometer. The viscosity‐average molecular weight (Mv) was calculated using the empirical relation ［η］ = 1.9 × 10^−^^7^ Mv^1.7^.

### 5.3 Morphological and Crystallinity Analysis

Polarized optical microscopy (POM) was performed using a Nikon Eclipse LV100POL microscope to observe birefringence and overall morphology. Particle size distribution was measured via laser diffraction using a HORIBA LA‐960 system. Sample density was determined using a helium pycnometer (Micromeritics AccuPyc II 1340).

Atomic force microscopy (AFM) was employed to characterize the surface morphology of poly(p‐benzamide) (PBA) whiskers. Samples were prepared by dispersing the whiskers in acetone via mild sonication, followed by drop‐casting onto cleaned sapphire substrates and drying under ambient conditions. AFM imaging was conducted in non‐contact mode using a Hitachi AFM5100N system equipped with silicon cantilevers. The resulting images revealed prismatic whiskers with typical dimensions of approximately 17 µm in length, 1 µm in width, and 0.5 µm in thickness.

### 5.4 Microcrystal Electron Diffraction (MicroED)

Micro ED Measurements Were Conducted Using an XtaLAB Synergy‐ED System (Rigaku Corporation/JEOL Corporation, Tokyo, Japan) operated at an accelerating voltage of 200 kV, with a dose rate of 0.01 electrons per Å^2^ per second and a tilt speed of 1°/s. Structural analysis was performed using Olex2 software (OlexSys, Durham, UK). Two sample preparation methods were employed: (1) PBA powder was dispersed on a glass substrate and directly transferred onto a TEM copper grid; (2) PBA powder was dissolved in acetone, followed by ultrasonic bath treatment to improve crystal dispersion. Crystals with sizes down to ∼500 nm were obtained.

### 5.5 Powder X‐Ray Diffraction and Electron Microscopy

Phase information of the PBA whisker powder was further confirmed via PXRD using a Rigaku Ultima diffractometer with Cu Kα radiation, operated at 40 kV—40 mA and room temperature. To correlate morphological features with crystallographic orientation, selected‐area electron diffraction (SAED) patterns were collected using a JEOL JEM‐2100F transmission electron microscope operated at 200 kV. Low‐dose conditions were employed with a small beam size (spot size 3), the electron beam was fully spread to a diameter of ∼10 µm to minimize the irradiation damage of the crystals during observation. The SAED patterns were taken with a selected‐area aperture ∼ 5 µm in diameter, with exposure time 0.3 s.

### 5.6 Thermal Analysis

Differential scanning calorimetry (DSC) and thermogravimetric analysis (TGA) were performed using a Mettler Toledo DSC1 and a Netzsch STA449 F1 instrument, respectively. DSC scans were conducted under nitrogen atmosphere from room temperature to 400°C at a heating/cooling rate of 5°C/min, for the pure PBA whisker samples and from room temperature up to 260°C at 5°C/min. for the PBA/nylon‐6 thin films, respectively. TGA measurements were carried out under argon and oxidative (Ar 80%:20% O_2_) atmospheres from 25°C up to 700°C at a heating/cooling rate of 10°C/min.

### 5.7 Thermal Expansion Measurements

The coefficient of thermal expansion (CTE) was measured using a Netzsch L75 dilatometer equipped with a Linear Variable Differential Transformer (LVDT). Powder samples were horizontally placed on the sample holder. Calibration was performed across the full temperature range to ensure accuracy.

### 5.8 Humidity Stability

Moisture uptake was evaluated by exposing samples to 50% relative humidity at 23°C for 24 h. Samples were weighed before and after exposure using a precision analytical balance. Prior to evaluation, samples were dried by vacuum oven.

### 5.9 Composite Preparation and Dispersion

Polyethersulfone (PES; SUMIKAEXCEL 4100P) was dissolved in dimethylformamide (DMF) at 10 wt.% and stirred at 400 rpm. PBA whiskers were added at 10 wt.% relative to PES. Nylon 6 was dissolved in hexafluoroisopropanol (HFIP) under identical conditions. Composite films were prepared by solvent evaporation. Cross‐sectional morphology was examined by polarized optical microscopy (POM) using a Nikon Eclipse LV100POL microscope in transmission mode after treating the film by dipping in 0.1 Mol KOH for 60 s.

### 5.10 Mechanical and Thermal Performance of Composites

CTE of composite films was measured using the same dilatometer setup as used for powder samples. Dynamic mechanical analysis (DMA) was performed using a TA Instruments Q800 in tension mode. Storage modulus and tan δ were recorded from 25°C to 250°C at a frequency of 1 Hz.

### 5.11 Small‐Angle X‐Ray Scattering (SAXS)

Small‐angle X‐ray scattering (SAXS) measurements were performed to investigate the internal chain arrangement and nanoscale structural features of poly(p‐benzamide) (PBA) whiskers. Samples polymerized at 340°C for 8 h were analyzed using a Xenocs Xeuss Pro instrument equipped with a RIGAKU FR‐X Cu Kα radiation source (λ = 0.154184 nm) and an EIGER2 R 1K 2D detector. The scattering profiles were collected over a q‐range of 0.1–3 nm^−^^1^ under ambient conditions. Data were corrected for background and normalized to the incident beam intensity. The absence of distinct scattering peaks in the whisker samples suggests a non‐lamellar, non‐periodic internal structure, which was further analyzed in the Discussion sections.

### 5.12 Second Harmonic Generation (SHG) Measurements

SHG activity was evaluated using a femtosecond Ti:sapphire laser (λ = 800 nm, pulse width ∼100 fs, repetition rate 80 MHz). PBA whiskers were drop‐cast onto glass substrates. SHG signals at 400 nm were collected in reflection mode and analyzed using a monochromator and photomultiplier tube. Reflected light was filtered by a monochromator and amplified by a photomultiplier tube to observe and analyze the 400 nm SHG signal.

## Author Contributions

S.O. conceived and supervised the project. S.O., J.L., and A.G. designed the experiments and analyzed the data. S.O. performed the synthesis, and J.L. and A.G. performed structural characterization of PBA whiskers. M.A. and D.H. conducted the micro‐electron diffraction and crystallographic analysis. J.W. provided theoretical insights and contributed to the interpretation of the experimental data. S.O. wrote the manuscript with input from all authors. All authors discussed the results and contributed to the final version of the manuscript.

## Conflicts of Interest

The authors declare no conflicts of interest.

## Supporting information




**Supporting File**: marc70204‐sup‐0001‐SuppMat.docx.

## Data Availability

The data that support the findings of this study are available in the supplementary material of this article.
