# Supplementary material for: Orthogonal Staggered Alignment of Molecular Chains in Aramid Whiskers: A Game‐Changer for Sustainable, High‐Performance Composites
Source: Macromol Rapid Commun. 2026 Jan 20;47(4):e00919. doi: 10.1002/marc.202500919 (PMC12922731; doi:10.1002/marc.202500919)
Supplement: Supplementary file 1 — Supporting File: marc70204‐sup‐0001‐SuppMat.docx. [file MARC-47-e00919-s001.docx]

**Supplementary Information**

**Experimental Condition**

**1. Reagents and Materials**

- **Monomer**: 4-acetamidobenzoic acid (ABA, purity ≥ 99%, Tokyo Chemical Industry Co., Ltd.)
- **Solvents**: Dibenzyltoluene (DBT, NEOSK-OIL 1400, Soken Tecnix Co., Ltd.), N,N-dimethylformamide (DMF, anhydrous 99.8%, Sigma-Aldrich Co., LLC), 1,1,1,3,3,3-hexafluoro-2-propanol (HFIP, purity ≥ 99%, Sigma-Aldrich Co., LLC), sulfuric acid (95.0%, FUJIFILM Wako Pure Chemical Corporation)
- **Polymers**: Polyethersulfone (SUMIKAEXCEL 4100P, Sumitomo Chemical Co., Ltd.), Nylon 6 pellets (Sigma-Aldrich Co., LLC), poly(oxybenzoate) (SUMIKASUPER E101, Sumitomo Chemical Co., Ltd.)

**2. Phase-Change Polymerization Conditions**

ABA (1.0 g) and DBT (100 g) were added to a 300 mL three-neck flask equipped with a thermometer, mechanical stirrer, and gas inlet/outlet tubes. The mixture was heated to 230 °C with stirring at approximately 400 rpm until the monomer was completely dissolved. Stirring was then stopped, and the temperature was further increased to 340 °C. Within several minutes, the reaction mixture became turbid and whiskers formed. The reaction was continued at 340 °C for 2–8 h under a slow stream of nitrogen. After cooling to room temperature, acetone was added to the mixture. The whiskers were collected by filtration, washed several times with hot acetone, and dried at 200 °C under reduced pressure for 10 h.

**3. Intrinsic Viscosity Measurement**

- A constant-temperature bath (TV-5NS; Thomas Scientific Instruments Co., Ltd., Japan) was used.
- An Ubbelohde capillary viscometer (No. 2) was employed for viscosity measurements.
- Sample concentrations were 0.5, 1.0, and 1.5 g/dL in 96% H₂SO₄.
- Measurements were conducted at 25 ± 0.1 °C.
- Prior to analysis, samples were dried at 100 °C for 1 h in a vacuum oven.
- Flow times were recorded in triplicate and averaged.
- Molecular weight was calculated using the Mark–Houwink equation:
- [η]=1.9×10^−7^Mv^1.7^

**4. Polarizing Microscope**

- Polarized optical microscopy (POM) was performed using Nikon Eclipse LV100POL and Keyence VHX‑S750 microscopes.

**5. Particle Size Distribution**

- Particle size distribution was measured using a laser diffraction/scattering particle size analyzer (LMS‑2000e, Seishin Enterprise Co., Ltd., Japan).
- The measurement is based on Mie scattering theory.
- Measurement range: 0.02–2000 μm.
- Dispersion medium: acetone.
- Ultrasonic dispersion time: 1 min.

**6. Crystallographic Analysis**

- XRD scan range: 5°–60° 2θ, step size: 0.02°, scan rate: 2°/min.
- µED zone‑axis alignment was verified using Kikuchi patterns.
- Unit cell refinement was performed using the SHELX software suite.

**7. Thermal Analysis Parameters**

- DSC: Heating and cooling rates of 5 °C/min for pure PBA whiskers (5 °C/min for PBA/nylon‑6 thin films), with an N₂ gas flow of 50 mL/min. Samples were sealed in pre‑weighed 40 µL Al pans with pierced lids.
- TGA: Heating and cooling rates of 10 °C/min, with a gas flow of 50 mL/min (argon or 20% O₂ / 80% Ar). Samples were lightly pressed into pre‑weighed 85 µL open alumina crucibles.
- The residual mass was normalized to the initial sample mass.

**8. Thermal Expansion Measurement**

- Atmosphere: Ar at 200 mL/min.
- Sample mass: 50 mg.
- Temperature range: 25–200 °C.
- Heating rate: 2 °C/min.
- The coefficient of thermal expansion was calculated as

  α = (1/L)(dL/dT),

and for discrete measurements,

  α ≈ (1/L_0_)(ΔL/ΔT),

where L₀ is the initial length at the reference temperature.

**9. Humidity Stability Test**

- Sample mass: Approximately 0.4 g.
- Pre‑treatment: Samples were placed in a temperature‑ and humidity‑controlled chamber prior to measurement.
- Environmental conditions: 23 ℃, 50% RH, 24 h.

**10. Composite Film Preparation**

- Films were cast on glass substrates and dried at 70 °C for 1 h.
- After peeling from the glass substrates, the films were dried at 100 °C for 1 h under vacuum.
- Film thickness: 20 µm.
- Cross‑sectional morphology was examined by polarized optical microscopy (POM) using a Nikon Eclipse LV100POL microscope in transmission mode after treating the films by immersing them in 0.1 M KOH for 60 s.

**11. DMA Conditions**

- Mode: Tensile.
- Temperature range: RT to 200 °C.
- Heating rate: 1 °C/min.
- Sample dimensions: 3.4 mm × 2.3 mm × 0.1 mm.
- Frequency: 1 Hz.
- Amplitude: 20 µm.
- Tg was determined from the peak of the tan δ curve.

**12. AFM measurement**

- Nominal spring constant: 40 N/m.
- Resonant frequency: 405 kHz.
- Height and phase images were acquired simultaneously at a scan rate of 1 Hz.

**13. SAXS measurement**

- Camera length: 500 mm.
- Exposure temperature: room temperature.
- Exposure time: 1 h.

**15. SHG Measurement**

- Laser power: 100 mW.
- Spot size: 100 µm.
- SHG intensity was normalized to quartz reference.

**Figures**

**Figure S1** Humidity stability of PBA whiskers.
Water uptake of PBA whiskers synthesized via 2 h and 8 h reactions (PBA-2HR and PBA-8HR), and POB whiskers, after 24 h exposure at 50% relative humidity and 23 °C. Both PBA whiskers exhibit <0.01 wt% water absorption.


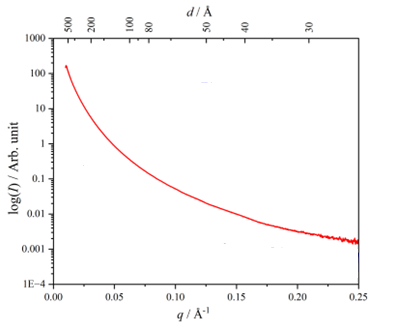


**Figure S2** Small-angle X-ray scattering (SAXS) profile of the PBA single crystal.
The scattering pattern reveals nanoscale structural features without detectable periodicity. The absence of characteristic peaks indicates that the crystal lacks a lamellar structure.

**Table**

**Table S1** Comparison of thermal stability, coefficient of thermal Expansion, and moisture absorption between PBA whiskers and cellulose nanofibers^21-23^
